# Circadian Biology and Phase Response: Fundamental Mechanisms and Clinical Applications

**DOI:** 10.3390/clockssleep8030048

**Published:** 2026-08-21

**Authors:** Malena L. Mul Fedele, Daniel P. Cardinali

**Affiliations:** 1 Chronophysiology Lab, Institute for Biomedical Research (BIOMED), Pontifical Catholic University of Argentina (UCA) and National Scientific and Technical Research Council (CONICET), Buenos Aires C1107AAZ, Argentina; 2 Faculty of Medical Sciences, Pontifical Catholic University of Argentina (UCA), Buenos Aires C1107AAZ, Argentina

**Keywords:** circadian rhythms, shift work, melatonin, light, synchronisation

## Abstract

The circadian clock, located in the mammalian hypothalamus, regulates biological rhythms with a period of approximately 24 h, influencing nearly all body functions. Its timing is synchronised daily by external cues, primarily light, which align internal rhythms with the environmental cycle. Through this entrainment, the circadian system orchestrates physiological processes such as the sleep–wake cycle, feeding behaviour, gene expression and body temperature regulation. Melatonin, secreted by the pineal gland, also plays a key role as a synchroniser by facilitating sleep onset. Changes in environmental time cues, such as those experienced by shift workers, can disrupt the body’s natural 24-h rhythms. This situation can lead to fatigue, significantly impacting accident rates and productivity, and, in the long term, to an increased risk of various health conditions. A Phase Response Curve (PRC) illustrates how the clock’s phase is affected by stimuli administered at different points in the circadian cycle. In particular, both light and melatonin can induce phase shifts, but the direction and magnitude of this shift depend on the timing of administration. Understanding the PRC enables the design of interventions to realign circadian rhythms and improve adaptation to shift work. This review explores the physiological and clinical effects of circadian disruption in shift workers and discusses strategies to mitigate its impact.

## 1. The Circadian Clock: How We Keep in Time with the Environment

The Earth’s rotation around its own axis generates a 24-h light–dark cycle. Moreover, the duration of daylight and darkness changes throughout the year as the Earth revolves around the Sun while maintaining a tilted rotational axis relative to the orbital plane. This dynamic has led living organisms to develop predictive mechanisms that enable them to anticipate and adapt to the daily light–dark cycle, which itself shifts with the seasons. Biological rhythms with a period close to 24 h (typically between 20 and 28 h) are known as circadian rhythms.

Almost all living organisms on Earth have developed a circadian clock, which in mammals is located in the brain, specifically in the suprachiasmatic nuclei (SCN) of the hypothalamus. These nuclei communicate with the retina through the retinohypothalamic tract (RHT) and are located above the optic chiasm [1]. This clock functions as a pacemaker, as it is capable of generating spontaneous oscillations autonomously and transmitting them to the rest of the body, allowing the organism to operate in an organized manner [2]. It can be entrained by external cues that set its time each day; and it is also capable of maintaining its oscillations under constant conditions, that is, without external time cues. The synchronisation process ensures that the circadian clock maintains a constant phase relationship with the environmental cycle. In this way, organisms can ‘prepare’ by anticipating what will happen in their environment, which has high adaptive value [3].

**Figure 1:**
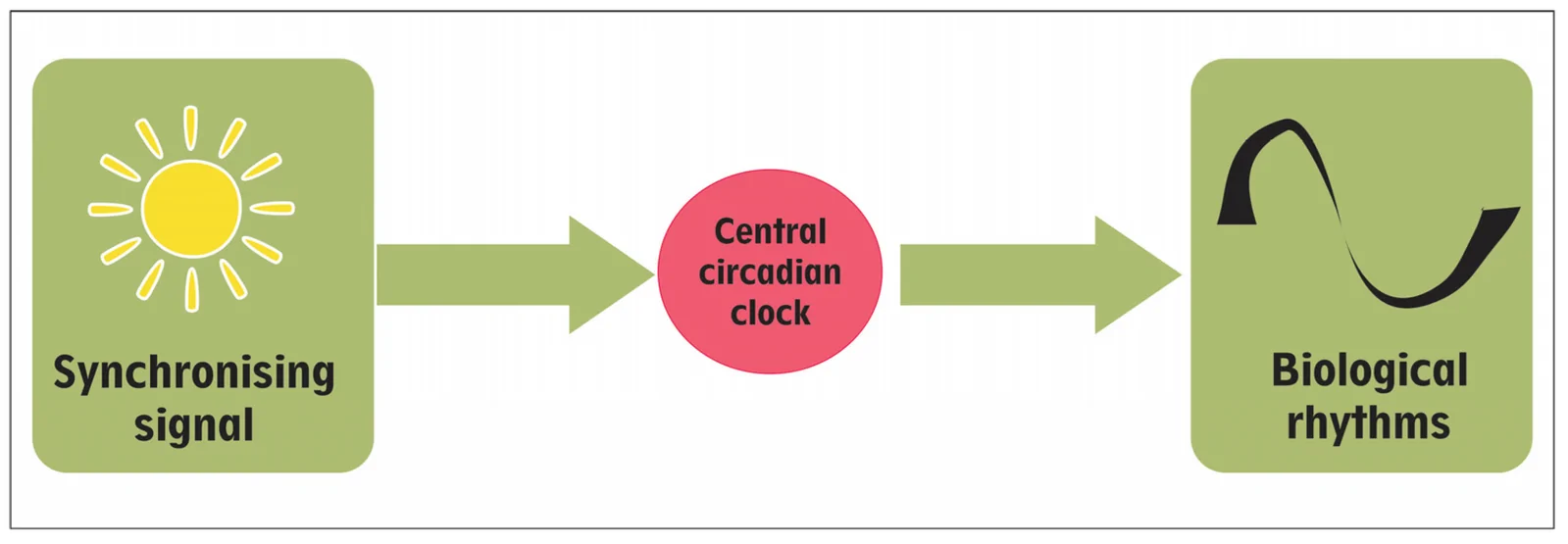
Simplified diagram of the circadian system (Eskinogram). A linear model that includes the components of the system. The endogenous central oscillator is influenced by an external synchronising agent through a process known as synchronisation. This leads to the generation of circadian rhythms in almost all physiological functions of the organism.

The most important synchronising signal is provided by light, but other stimuli, such as temperature, food, and social interactions, can also entrain the circadian clock. Those external cues that can synchronise the circadian clock are also known as zeitgebers. This process establishes a structured framework: the entrainment or “synchronisation” of the central circadian clock by the environment generates rhythmicity in physiological functions, such as the sleep–wake cycle, feeding behaviour, and body temperature regulation (Figure 1) [4]. Without the influence of these external synchronisers, the period of these oscillations tends to be longer than 24 h.

## 2. Neurochemical and Anatomical Architecture of the Suprachiasmatic Nuclei

**Figure 2:**
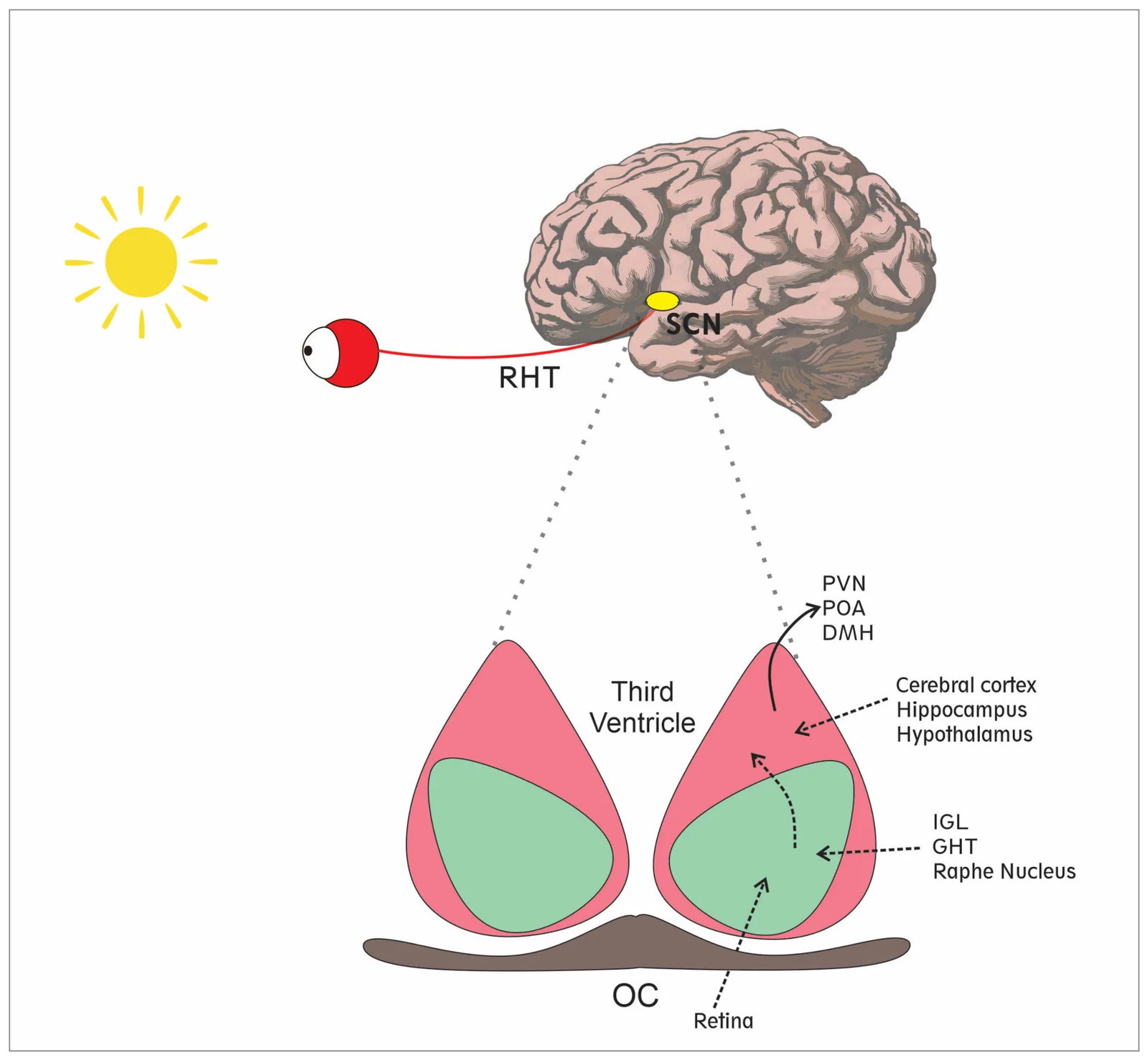
Location and neuroanatomical characteristics of the SCN. Diagram of the arrangement of both regions of the SCN: Shell and Core. Dashed arrows indicate afferent connections to both regions, while solid arrows indicate efferent connections. OC: optic chiasm. IGL: intergeniculate leaflet. GHT: geniculohypothalamic tract. PVN: paraventricular nucleus. POA: preoptic area. DMH: dorsomedial hypothalamus.

Structurally, two anatomical subdivisions can be identified in each SCN, which differ in terms of cytochemistry: the dorsomedial region, or Shell, and the ventrolateral region, or Core (Figure 2). The Core area has a high cell density and receives direct input from the retina via the RHT, and secondary visual input from the intergeniculate leaflet and the geniculohypothalamic tract, as well as from the raphe nucleus. The Shell region partially surrounds the Core and receives input from it, as well as from the cerebral cortex, hippocampus, and other hypothalamic regions [5]. These observations support the idea that the Core primarily receives photic signals (from the retina) critical for synchronising the clock to the light–dark cycle, while the Shell receives projections from the Core related to non-photic modulatory inputs. The neurons in the Shell are thought to be modulated by these inputs and are therefore responsible for autonomously regulating the outputs of the clock according to the signals received.

As previously mentioned, neurons in the Shell project to other areas of the hypothalamus, such as the paraventricular nucleus (PVN), the preoptic area, and the dorsomedial hypothalamus (DMH). These regions are involved in endocrine, immune, thermoregulatory, and autonomic control, and they help regulate rhythms in peripheral tissues, acting as output pathways of the central clock [6]. Through these projections, the SCN exerts its circadian influence via both hypothalamic (endocrine) and extrahypothalamic (behavioural) pathways. Specifically, these effects are directed towards:

- neuroendocrine neurons in the PVN;
- autonomic neurons in the PVN;
- hypothalamic structures associated with sleep, such as the ventrolateral preoptic area (VLPO);
- other hypothalamic regions (sub-PVN, DMH, medial preoptic area), which serve as intermediaries between the SCN and autonomic and neuroendocrine neurons;
- extrahypothalamic structures (lateral geniculate body, paraventricular thalamic nucleus) involved in synchronizing hypothalamic pathways and locomotor activity [7].

The neurons in each subregion are distinguished by their distinct neurochemical content. Most neurons in the Core contain vasoactive intestinal peptide, while a smaller proportion contains gastrin-releasing peptide, neurotensin, and calretinin. In the Shell region, neurons contain arginine vasopressin, cholecystokinin, prokineticin 2, calbindin, angiotensin II, and met-enkephalin [5,8,9]. Additionally, in most SCN neurons, neuropeptides are co-localized with the neurotransmitter gamma-aminobutyric acid (GABA), and almost all synapses within the SCN are GABAergic [9,10]. Electrophysiological evidence has also shown that glutamate can act as a neurotransmitter in the SCN’s efferent pathways [9]. Another characteristic of the mammalian SCN is the high abundance of glial cells, particularly astrocytes [11].

## 3. Molecular Mechanisms of the Circadian Clock

**Figure 3:**
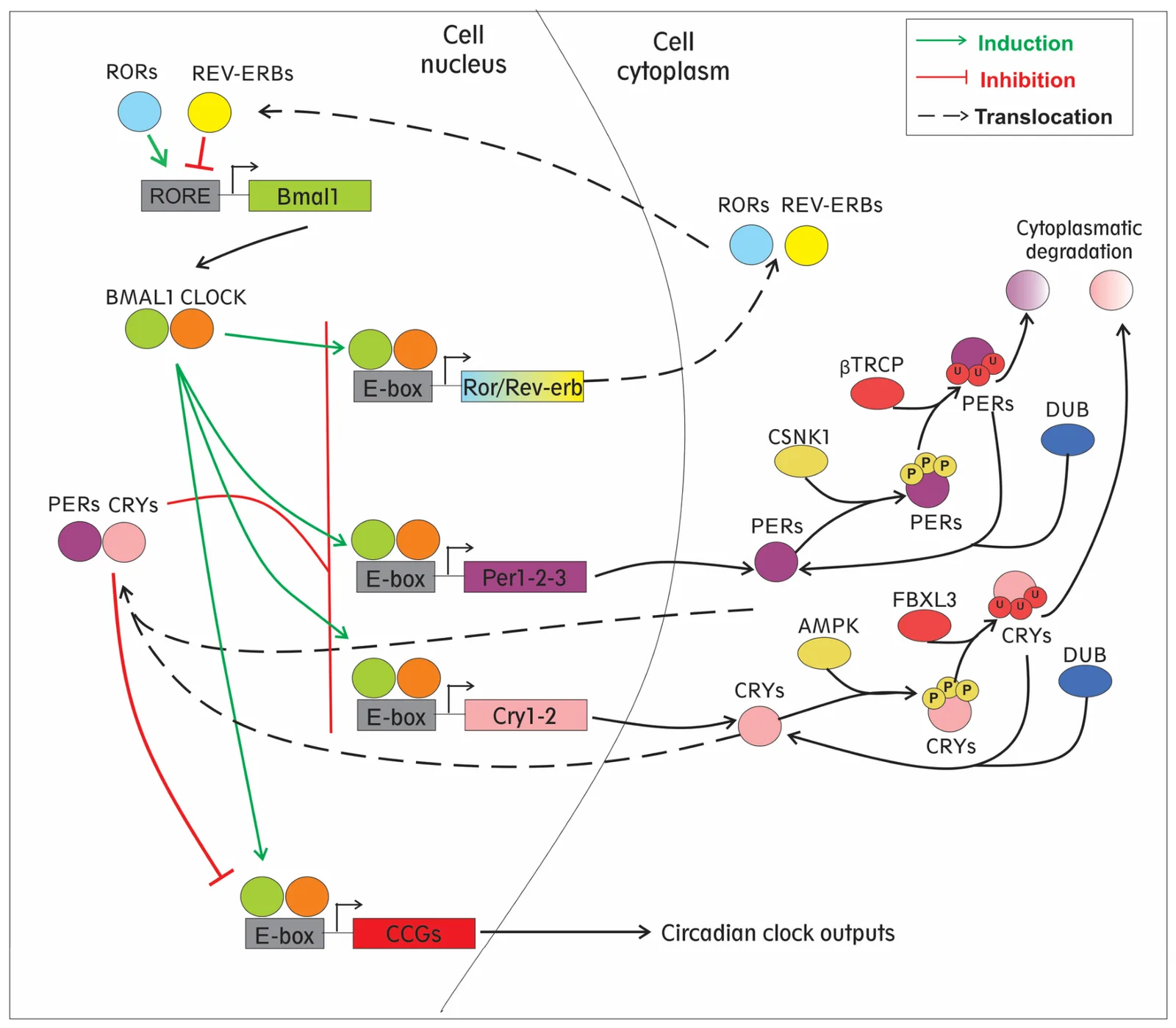
Molecular components of the mammalian circadian clock. The core feedback loop is driven by CLOCK and BMAL1 proteins. These proteins heterodimerize and initiate the transcription of the *Period* (*Per* 1, *2*, and *3*) and *Cryptochrome* (*Cry 1* and *2*) genes, as well as other *clock-controlled* genes (CCGs). The negative feedback loop is carried out by heterodimers formed by PER (1 or 2) and CRY (1 or 2), which translocate into the nucleus, heterodimerize, and inhibit their own transcription and that of other genes containing E-box elements, by binding to the CLOCK-BMAL1 heterodimer. In addition, the CLOCK-BMAL1 heterodimer activates the transcription of the nuclear receptors ROR and REV-ERB, which bind to the RORE sequence and activate or inhibit *Bmal1* transcription. Casein kinases 1 epsilon and 1 delta (CSNK1), as well as 5′ adenosine monophosphate-activated protein kinase (AMPK), can phosphorylate PER and CRY proteins. Subsequently, the F-box-type E3 ligases β-TrCP and FBXL3 recognize phosphorylated PER and CRY proteins, targeting them for polyubiquitination and eventual degradation via the proteasome. There are deubiquitinases (DUB) that can deubiquitinate PER and CRY proteins, increasing their stability and enhancing their nuclear translocation. Red lines represent repression mechanisms, green lines indicate activation, and black dashed lines indicate translocation.

The autonomous nature of SCN cells has driven research into the cellular and molecular processes underlying the clock mechanism. Its core components are genes whose protein products are essential for the autonomous generation and regulation of circadian rhythms [12]. It is now known that the molecular clock is composed of interconnected positive and negative transcriptional and translational feedback loops (Figure 3). In the main feedback loop, transcription factors of the bHLH-PAS (basic helix–loop–helix–Period–Arnt–Single-minded) family are involved—specifically a gene called *Clock* and another gene called *Bmal1* (*Brain and muscle ARNT-like 1*). The protein products of these genes form heterodimers that initiate the transcription of genes containing the regulatory E-box sequence. These include the *Period* (*Per*) *1*, *2*, and *3* genes; *Cryptochrome* (*Cry*) *1* and *2*; *Rev-erbα/β* and *Rorα/β/γ*; and *clock-controlled genes* (*CCGs*). The negative feedback loop is carried out by the heterodimer formed by PER and CRY proteins, which translocate to the nucleus, form heterodimers, and interact with the CLOCK-BMAL1 complex, inhibiting its activity. In doing so, they also repress their own transcription and that of the CCGs. While CCGs do not participate in the oscillatory mechanism itself, they are involved in generating biological rhythms, as their products represent the outputs of the clock [13].

The CLOCK-BMAL1 heterodimer also generates additional feedback loops involving the retinoic acid-related nuclear receptor families ROR and REV-ERB. Members of these receptor families compete for binding to ROREs (retinoic acid-related orphan receptor response elements) located in the *Bmal1* promoter, thereby activating or inhibiting its transcription, respectively. These proteins, therefore, regulate the expression of *Bmal1* [12,13].

The feedback loops take approximately 24 h to complete a cycle, thus constituting an autonomous molecular circadian clock. This molecular circadian clock operates in virtually all cells of the body to regulate the expression of nearly half of the genome [14]. Additionally, this mechanism is regulated by post-transcriptional modifications such as phosphorylation and ubiquitination. Phosphorylation is carried out by casein kinases 1 epsilon and 1 delta (CK1ε and CK1δ), as well as 5′ adenosine monophosphate-activated protein kinase (AMPK), which phosphorylate PER and CRY proteins to promote their degradation via ubiquitination. F-box-type E3 ligases, β-TrCP and FBXL3, recognize phosphorylated PER or CRY proteins and promote their polyubiquitination and subsequent proteasomal degradation (Figure 3). These processes contribute to the precision of the circadian clock [15,16,17].

## 4. From Light to Physiology: Central and Peripheral Circadian Synchronisation

As mentioned earlier, photic information reaches the SCN through the RHT, which carries axons from photosensitive retinal ganglion cells containing the photopigment melanopsin and releasing the neurotransmitter glutamate. This neurotransmitter causes membrane depolarization, leading to calcium influx that activates a kinase complex (CaMK, MAPK, and PKA). These kinases phosphorylate the CRE-binding protein, a calcium/cAMP response element-binding protein, which becomes activated and induces the expression of genes containing this element [18].

This suggests that the light perceived by retinal ganglion cells generates a signal sent to the SCN, which then signals peripheral oscillators to regulate circadian physiology. This information is transmitted to specific areas of the basal hypothalamus, which control the two main communication pathways of the body: the endocrine system and the autonomic nervous system [19,20,21]. Peripheral clocks regulate processes specific to each tissue, such as metabolism, hormone release, immune responses, and cell repair. While they are synchronised by the central pacemaker located in the suprachiasmatic nucleus through neural and hormonal pathways, these peripheral oscillators can also be adjusted by local time cues, including feeding schedules, temperature changes, physical activity, and glucocorticoid levels [22,23,24]. In response to this pathway, nearly all physiological processes exhibit a 24-h rhythm. However, the timing of the peak of these rhythms can vary. This gives rise to a sequence of peak values for each rhythm with a specific spacing (phase map), revealing various patterns of cause-and-effect relationships among the body’s functions, both molecular and behavioural [25].

Thus, the circadian system consists of (a) a hypothalamic pacemaker (the SCN); (b) a series of physiological outputs under SCN control; and (c) molecular clocks present in all tissues and organs.

## 5. Circadian Rhythm Analysis

**Figure 4:**
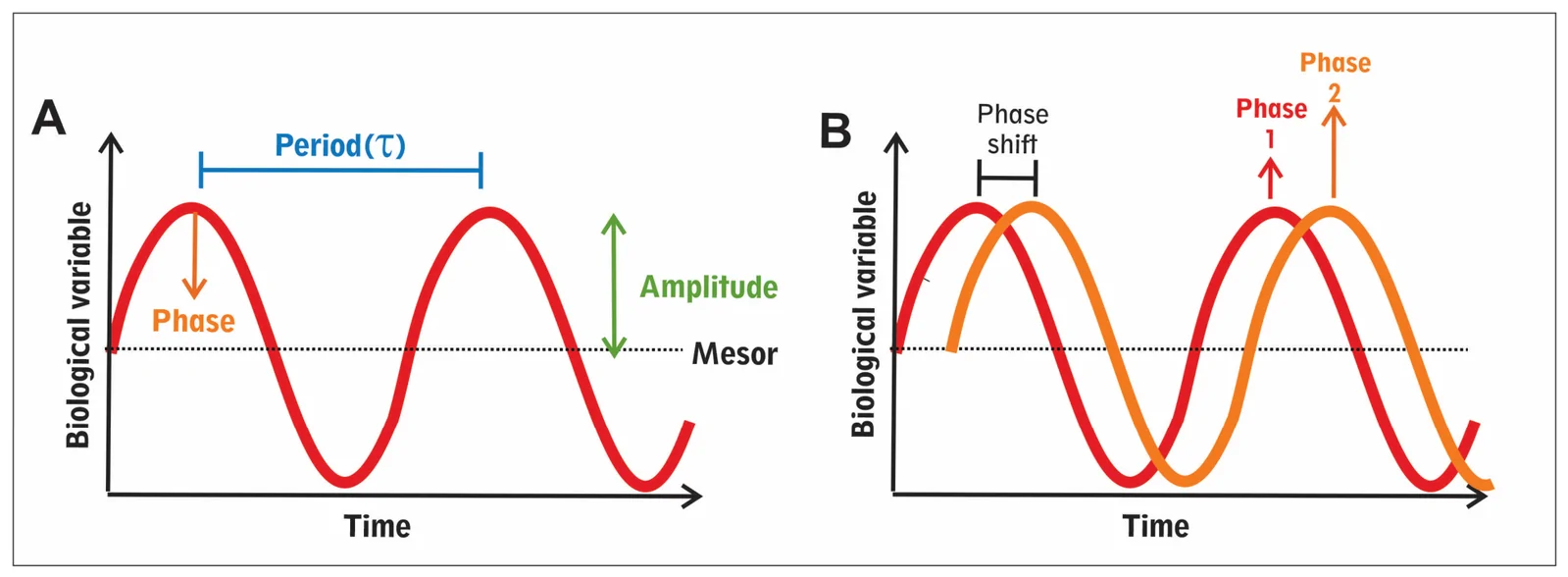
Characteristic parameters of a rhythm. (**A**) Representation of the oscillation of a rhythmic variable over time. The four key parameters of a rhythm are indicated: period, mesor, amplitude, and phase. (**B**) Two oscillations with different phases are shown. Any point on the rhythm can be used as a phase marker.

Rhythms can be analyzed by fitting the data to a sinusoidal function, from which four fundamental parameters are defined: the Period (τ, tau), which is the time it takes for one complete oscillation to repeat; the Mesor, which is the mean value the variable takes over the rhythm; the Amplitude, which is the difference between the maximum value and the mesor; and the Phase (Ψ), which corresponds to the time at which the variable reaches a specific value (Figure 4A). Any point on the rhythm can be taken as a reference value or phase marker [26]. These parameters can be used to compare or study alterations in rhythms.

Different stimuli can cause a phase shift in the circadian clock (Figure 4B). A phase shift refers to a change in the timing of a circadian rhythm. This shift does not necessarily alter the length of the rhythm but instead moves it earlier or later than usual. A phase advance occurs when the rhythm shifts earlier, while a phase delay occurs when it shifts later. Phase shifts can be measured using markers such as the onset of melatonin production, core body temperature, and sleep timing.

**Figure 5:**
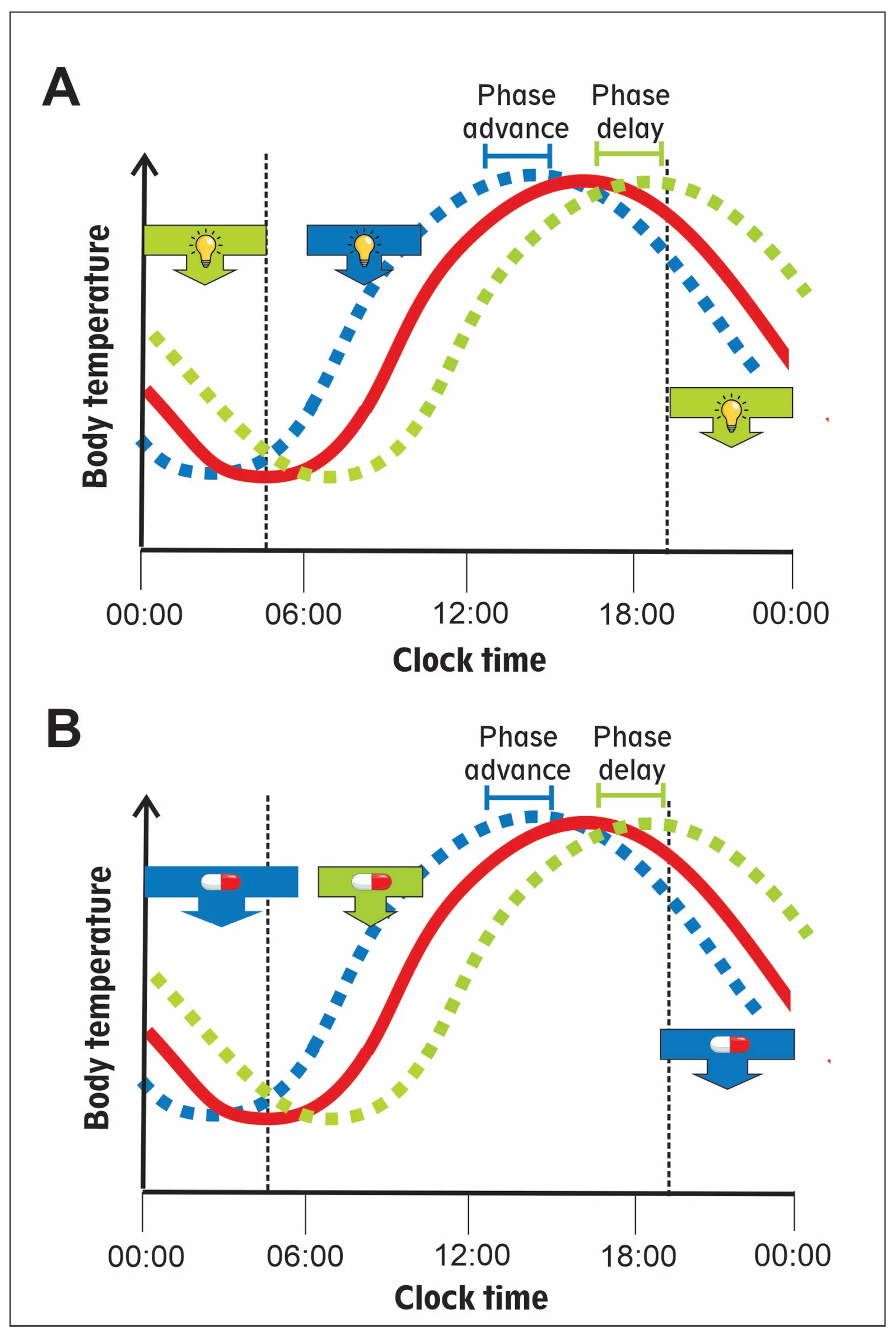
Phase shift in the core body temperature circadian rhythm induced by light or melatonin administration. The baseline core body temperature circadian rhythm is represented by the red line. (**A**) If a light pulse is administered during the evening or early part of the night, it induces a phase delay of the core body temperature circadian rhythm (green line), while if it is administered during the second half of the night or early morning, it induces a phase advance (blue line). (**B**) Melatonin administered in the evening or early part of the night induces a phase advance in the core body temperature circadian rhythm (blue line); if it is administered during the early morning, it induces a phase delay (green line). The vertical dashed lines indicate the daytime interval (approximately 07:00 to 19:00). The horizontal extent of the blue and green arrows represents the time window during which administration of the stimulus produces a phase advance or phase delay, respectively. Adapted with permission from [28].

As mentioned earlier, light is the most important circadian synchroniser, and as such, it can shift the phase of a circadian rhythm. In humans, if a light pulse is administered during the evening or early part of the night, it induces a phase delay, meaning the circadian clock is slowed down and sleep begins later on subsequent days. In contrast, if the light pulse is administered during the second half of the night or early morning, it induces a phase advance, meaning the clock is sped up and sleep begins earlier on subsequent days (Figure 5A) [27].

## 6. Melatonin as a Circadian Zeitgeber

Another major synchroniser of the circadian clock is melatonin, which begins to be released from the pineal gland at the end of the afternoon, at approximately 18:00 h. This hormone reduces the electrical activity via MT1 and MT2 melatonin receptors located on SCN cells, and therefore their ability to neutralize sleep pressure (propensity to sleep), which is crucial for sleep induction [28,29].

The circadian rhythm of melatonin secretion plays an important role in the regulation of the sleep–wake cycle in both healthy and blind individuals. Plasma melatonin levels start to rise before sleep onset and reach their peak during the first part of the night [30]. Thus, the circadian rhythm of melatonin in plasma or saliva, or its metabolite 6-sulfatoxymelatonin (aMT6s) in urine, can serve as an indicator of circadian clock function [31].

The projections from the SCN, which control the daily rhythm of melatonin synthesis, inhibit neurons in the paraventricular zone of the hypothalamus. From this region, a multisynaptic pathway begins, involving the medial forebrain bundle, the reticular formation, the intermediolateral cell column of the cervical spinal cord, the superior cervical ganglia, and postganglionic sympathetic fibers that terminate near the pineal gland, where melatonin synthesis takes place [28].

Exogenous melatonin acts as a chronobiotic signal capable of causing a phase shift in the human circadian system in a direction generally opposite to that induced by light [32]. This hormone induces phase advances when administered in the evening or early part of the night, and phase delays when administered in the early morning (Figure 5B). It is generally considered a non-photic stimulus, as it can reset the circadian clock through signalling pathways that differ from those activated by photic (light) stimuli [4]. Experimental evidence indicates that melatonin can cause a phase shift in the human circadian clock through activation of MT1 and MT2 receptors within the SCN. MT1 receptor activation is thought to suppress SCN neuronal firing, whereas MT2 receptors appear to play a more prominent role in circadian phase resetting [29]. Moreover, although direct actions on the electrical and metabolic activity of the SCN are considered the principal mechanism underlying melatonin-induced phase shifts, the downstream mechanisms involved in non-photic circadian resetting, together with the contribution of its sleep-promoting properties, remain incompletely understood [4,29,33].

Bright light can suppress melatonin production [34]. However, other external influences that can alter circadian rhythms, such as carbohydrate intake, do not affect melatonin secretion [35]. Thus, dim light melatonin onset (DLMO), the time at which the body begins producing melatonin under dim light conditions (typically around 10 lux), is one of the most accurate markers of circadian phase. It can also be used to determine whether an individual is synchronised with external cues, such as the light–dark cycle [31].

## 7. Phase Response Curves: Temporal Dynamics of Circadian Entrainment

The Phase Response Curve (PRC) illustrates how the clock’s phase is affected by stimuli administered at different points in the circadian cycle. It shows the magnitude and direction of the phase shift, and it can typically contain a delay and/or an advance part, and a zone where there are no substantial changes in response to the stimulus. It is an intrinsic property of the circadian oscillator, and its final shape is characteristic of each stimulus and species, including humans [27,36]. It typically illustrates the circadian clock’s sensitivity and how various zeitgebers influence circadian rhythms.

When we study the effects of light on the clock phase, this results in a photic PRC, where the circadian timing of light administration is represented on the *x*-axis, and the phase shift in the rhythm is shown on the *y*-axis. Other types of stimuli, which can also cause a phase shift in the circadian clock, give rise to non-photic PRCs [4]. It is important to note that phase advances are plotted with positive values, and delays are plotted with negative values.

Moreover, depending on the strength of the resetting stimulus and the state of the circadian oscillator, PRCs may be classified as type 1, characterised by relatively small phase shifts and a broad transition between delays and advances, or type 0, characterised by large phase shifts and an abrupt transition between delays and advances. In humans, the PRCs obtained under physiological conditions with light or melatonin generally correspond to type 1 resetting [37].

**Figure 6:**
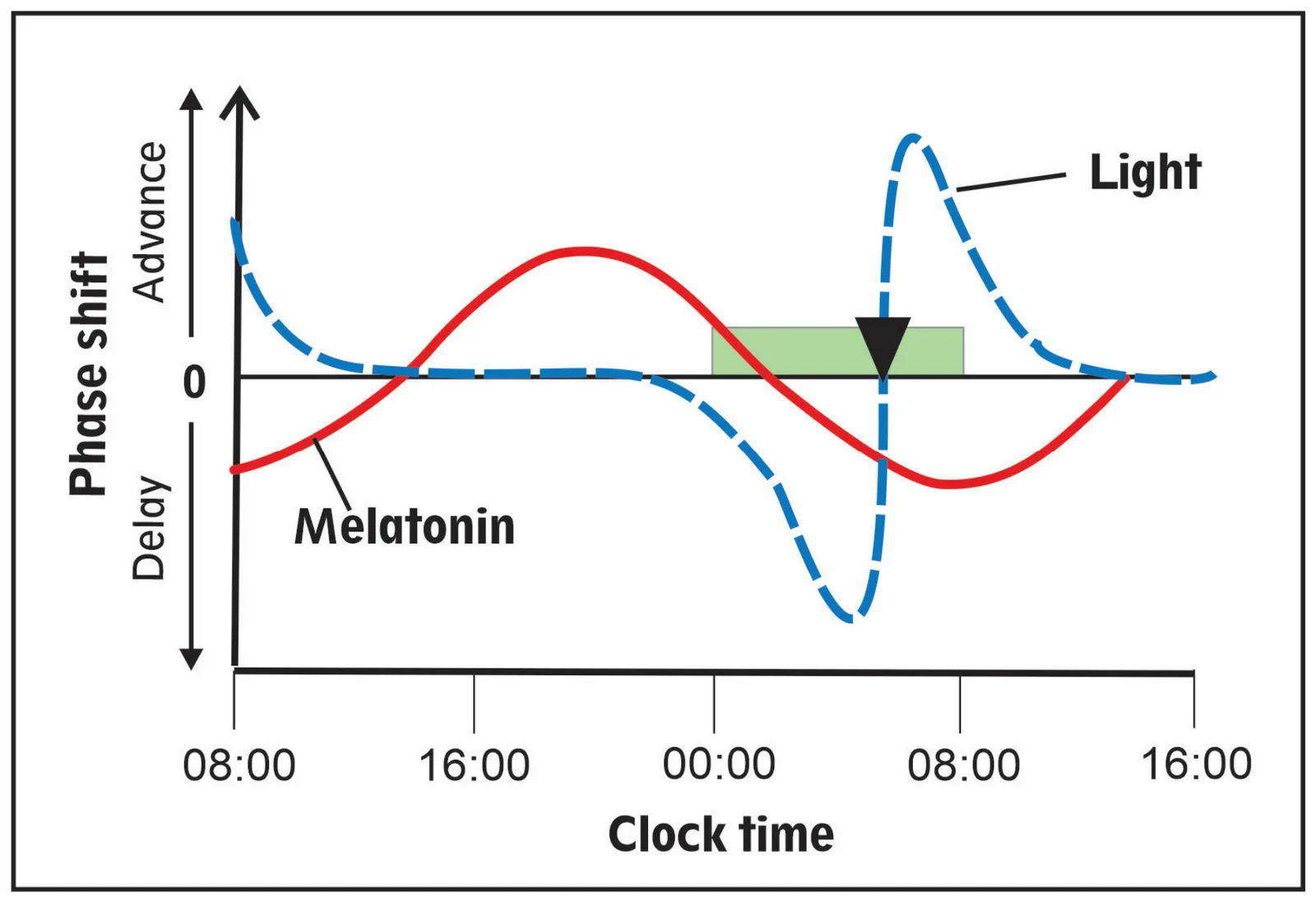
Schematic phase response curve (PRC) for light (blue dashed line) and exogenous melatonin (red). It depicts the direction, relative magnitude of the phase shift and phase relations. The *x*-axis represents the clock time of stimulus administration over a 24-h period for an individual with a nocturnal sleep schedule. The *y*-axis shows the resulting phase shift in the core body temperature circadian rhythm. Positive values represent phase advances, and negative values indicate phase delays. The filled triangle indicates the approximate timing of the minimum of the core body temperature rhythm, which serves as the reference circadian phase marker (crossover point) for both PRCs. The green rectangle represents the habitual timing and duration of sleep. The curves are schematic and illustrate the relative timing and magnitude of phase shifts rather than exact quantitative values. Modified with permission from [28].

In humans, PRCs have been established under carefully controlled laboratory conditions designed to minimize the influence of external zeitgebers other than the experimental stimulus. Participants are typically maintained on stable sleep–wake schedules and under dim-light conditions, while circadian phase is assessed using physiological markers such as the DLMO or the minimum of the core body temperature rhythm. A light pulse or melatonin is administered at different circadian phases, and the resulting phase shifts are quantified by comparing the circadian physiological marker before and after the intervention [27,38]. Figure 6 illustrates the PRCs for both light (in blue) and exogenous melatonin (in red) administration. The minimum of the core body temperature rhythm (CBTmin) serves as the physiological reference point (crossover point) of both curves. For illustrative purposes, the *x*-axis expresses clock time assuming a nocturnal sleep schedule, although the phase-shifting effects depend on circadian phase rather than clock time itself. When light exposure occurs before the CBTmin, it results in a delay of the core body temperature circadian rhythm, whereas light administered after this point leads to a phase advance. The most significant phase shifts occur when light is applied near the moment when the CBTmin occurs [39]. Under well-controlled conditions, exposure to bright light can cause phase delays of about 2.5 to 3 h per day and phase advances of around 1.5 to 2 h per day [37]. As depicted, the human melatonin PRC has been characterised in several experimental studies and is approximately opposite in phase to the light PRC, although not perfectly symmetrical [32,38] (Figure 6). This relationship provides the physiological basis for the combined use of timed light exposure and melatonin to facilitate circadian adaptation. When melatonin is administered during the day, it results in an advance of the body temperature rhythm, whereas melatonin administered near and after this point leads to a phase delay. Most studies have used low pharmacological doses of melatonin (typically 0.5–3 mg), which are sufficient to induce measurable phase shifts [32,38,40]. In the melatonin PRC, the first half of the night reflects the zone of reduced responsiveness, whereas in the light PRC, the phase shifting responses are reduced during the day.

## 8. Living in a 24/7 Society: Consequences for the Circadian System

**Figure 7:**
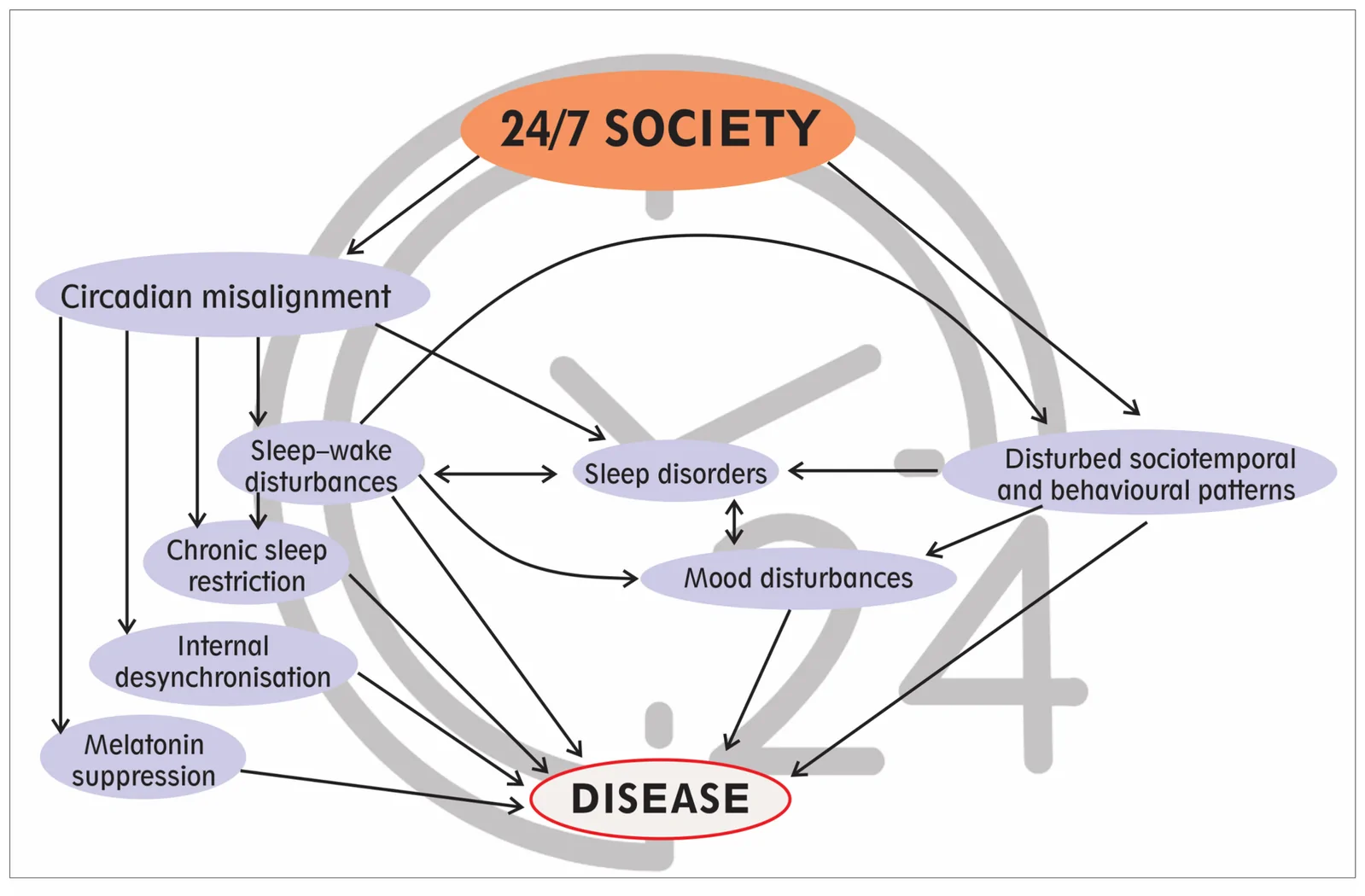
Impact of the 24/7 society. In today’s 24/7 society, light exposure no longer reliably signals the time of day. Disruptions to environmental time cues can lead to chronodisruption, affecting the sleep–wake cycle, melatonin production, and the function of peripheral clocks. In the short term, these disturbances can cause fatigue, increasing the risk of accidents and reducing productivity. Over the long term, circadian disruption and shortened sleep duration are strongly linked to a higher risk of disease. Modified with permission from [28].

When the clock is properly synchronised with the environment, it promotes sleep and anabolic functions—such as immune function and hormone secretion—at night, while supporting wakefulness and catabolic functions—such as feeding and physical activity—during the day [41]. Our modern “24/7 society” requires non-standard working conditions, such as shift work. As a result, photic signalling no longer reliably encodes the time of day. Changes in environmental time cues (zeitgebers) can disrupt the body’s natural 24-h rhythms. Exposure to artificial light from evening through early morning, collectively known as artificial light at night (ALAN), strongly suppresses the activity of sleep-promoting neurons in the hypothalamus and stimulates brain systems that support wakefulness. Since melatonin secretion depends on dark-phase cues, exposure to light at inappropriate times can disrupt its circadian rhythm. Like natural light, ALAN suppresses melatonin secretion in humans [34,42], which plays a key role in aligning our internal clock and triggering the onset of sleep [43]. For example, attempting to sleep around 6:00 or 7:00 p.m. typically results in only a short nap of 1–2 h. In contrast, delaying sleep onset to between 9:00 and 10:00 p.m. usually allows for a longer, uninterrupted 6–8-h sleep. This significant rise in the drive to sleep is due to melatonin’s role in dampening the wakefulness-promoting signals from the SCN. Light exposure during the evening interferes with melatonin release, thereby decreasing drowsiness, increasing alertness, and disrupting the ability to fall and stay asleep (Figure 7).

We define non-standard work schedules as those that involve working more than eight hours per day or 40 h per week and/or between 9:00 pm and 9:00 am. It is estimated that in developed countries, between 20% and 25% of workers follow non-standard work schedules [44,45]. In shift work, the activity/rest cycle becomes misaligned with the light/dark cycle, leading to phase shifts that create challenges for workers adapting to schedules that conflict with both environmental cues (like light and other zeitgebers) and their internal biological clock [46].

## 9. Chronodisruption Caused by Shift Work and Light at Night

**Figure 8:**
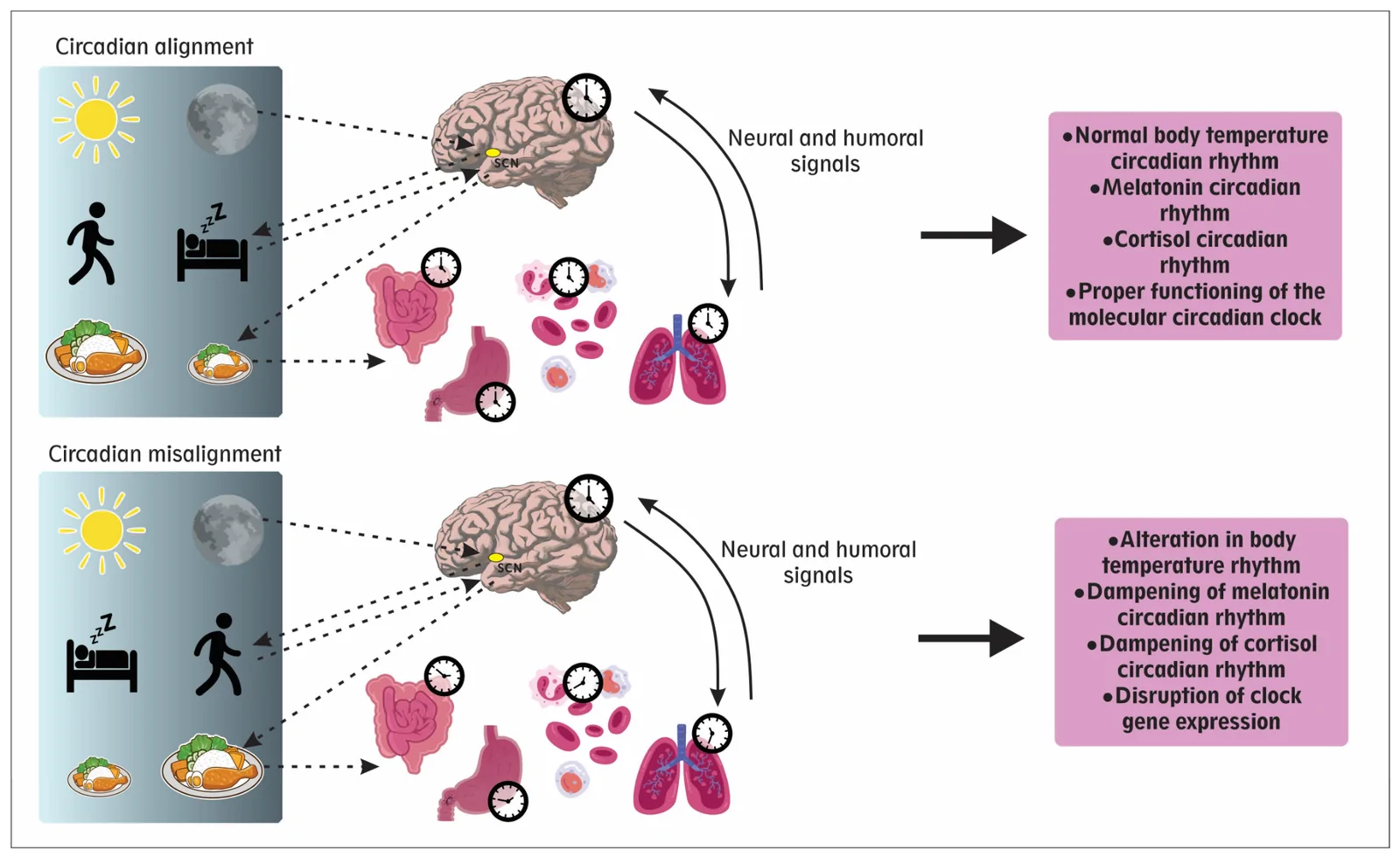
Effect of circadian misalignment. Misalignment of external cues, such as light exposure, meal timing, and the sleep–wake cycle, can lead to chronodisruption. This disruption may affect various components of the circadian system, including the central clock, the signals it sends to regulate other physiological systems, and the functioning of peripheral clocks. Such alterations can be observed by examining markers like the core body temperature rhythm, melatonin and cortisol secretion patterns, or clock gene expression, among others.

As previously mentioned, nearly all physiological processes follow a circadian “phase map” characterised by a predictable pattern of peaks and troughs. These phase maps are highly responsive to environmental influences and can be temporarily altered by disruptions in timing, such as shift work or the impact of acute or chronic illnesses, regardless of how severe they are. The term chronodisruption refers to alterations in the amplitude and phase of circadian rhythms, which are commonly seen alongside many acute and chronic medical conditions. Such disruptions may result from dysfunction in one or more elements of the circadian system, whether within the central clock itself, in the signals it sends to regulate other systems, in the presence or effectiveness of environmental time cues (synchronisers), or in the way zeitgebers communicate with the biological clock (Figure 8). In clinical settings, pinpointing the exact source of these disturbances is rare, and in most cases, the causes are complex and involve multiple factors [47,48,49,50].

Nocturnal and shift workers experience abrupt changes in their sleep–wake and light–dark cycles and are especially affected by ALAN. The timing of the melatonin rhythm reflects the state of the circadian clock, providing information about its phase (i.e., the internal clock’s alignment with external time) and its amplitude [51]. Some studies have found that night-shift workers have a reduction in the amplitude of the circadian rhythm of aMT6s, the major urinary metabolite of melatonin, compared to day workers [52]; those levels remained low even when the workers slept at night [53]. Moreover, a meta-analysis showed that night-shift workers, especially those with a fixed schedule, had lower levels of aMT6s [54]. Also, some studies found a relationship between chronotype (diurnal preference for activity) and melatonin levels among shift workers, suggesting that better alignment of chronotype with the work schedule produces less disruption of the melatonin circadian rhythm [52,55].

## 10. Physiological and Clinical Impacts of Shift Work

Circadian clock desynchronisation has both short- and long-term consequences. In the short term, it can lead to fatigue, significantly impacting accident rates and productivity. In the long term, circadian rhythm disruption and reduced sleep duration—caused by night work or rotating shifts—are strongly associated with an increased risk of various health conditions, including obesity, type 2 diabetes, metabolic syndrome, cardiovascular diseases, and certain types of cancer (Figure 7) [56,57,58,59,60]. Moreover, due to the substantial body of evidence, in 2007, the International Agency for Research on Cancer from the World Health Organization classified shift work as a probable human carcinogen (2A) [61], which appears to be directly related to the disruption of the circadian clock’s molecular machinery [62].

Studies have shown that women who work rotating night shifts over long periods face a moderately higher risk of developing breast cancer [63], a pattern also observed among female airline cabin crew [64]. Melatonin levels are generally low in cancer patients, with few exceptions [28], suggesting ongoing circadian disruption, as circulating melatonin reflects the functional state of the SCN. A widely supported theory is that reduced melatonin secretion contributes to cancer development under chronodisruption. This light–melatonin–cancer hypothesis has been supported by different experimental studies. For example, in a mouse melanoma model, circadian disruption was shown to accelerate tumor growth and disrupt immune and gene expression rhythms, fostering a tumor-promoting and proliferative environment [65]. In another study, rats exposed to continuous light showed a sevenfold increase in tumor growth compared to those maintained under standard light–dark cycles [66]. This was linked to elevated linoleic acid uptake and metabolism, which was attributed to suppressed melatonin signaling, that typically inhibits this process at night. Tumors exposed to nocturnal, melatonin-rich human blood exhibited significantly reduced proliferation and linoleic acid metabolism compared to those perfused with daytime, melatonin-deficient blood [67]. In prostate cancer models, increasing nighttime melatonin levels, by exposing rats to blue light during the day, significantly decreased cancer metabolic and proliferative activity [68]. Overall, these findings may help explain the higher cancer risk observed in shift workers [69,70]. Additionally, it was observed that melatonin rhythm disruption can be linked to increased lung cancer risk [71]. A large body of experimental and clinical research supports the anti-cancer (oncostatic) properties of melatonin [28,72]. However, despite the compelling experimental evidence, the clinical relevance of these findings remains uncertain. Human studies are heterogeneous with respect to tumor type, treatment protocols, melatonin dose, and study design, and recent systematic reviews have concluded that current evidence is insufficient to establish the efficacy of melatonin as an adjunctive anticancer therapy [73]. Therefore, although melatonin suppression represents a plausible biological mechanism linking circadian disruption and cancer, its precise contribution to cancer development and progression remains to be fully established.

**Figure 9:**
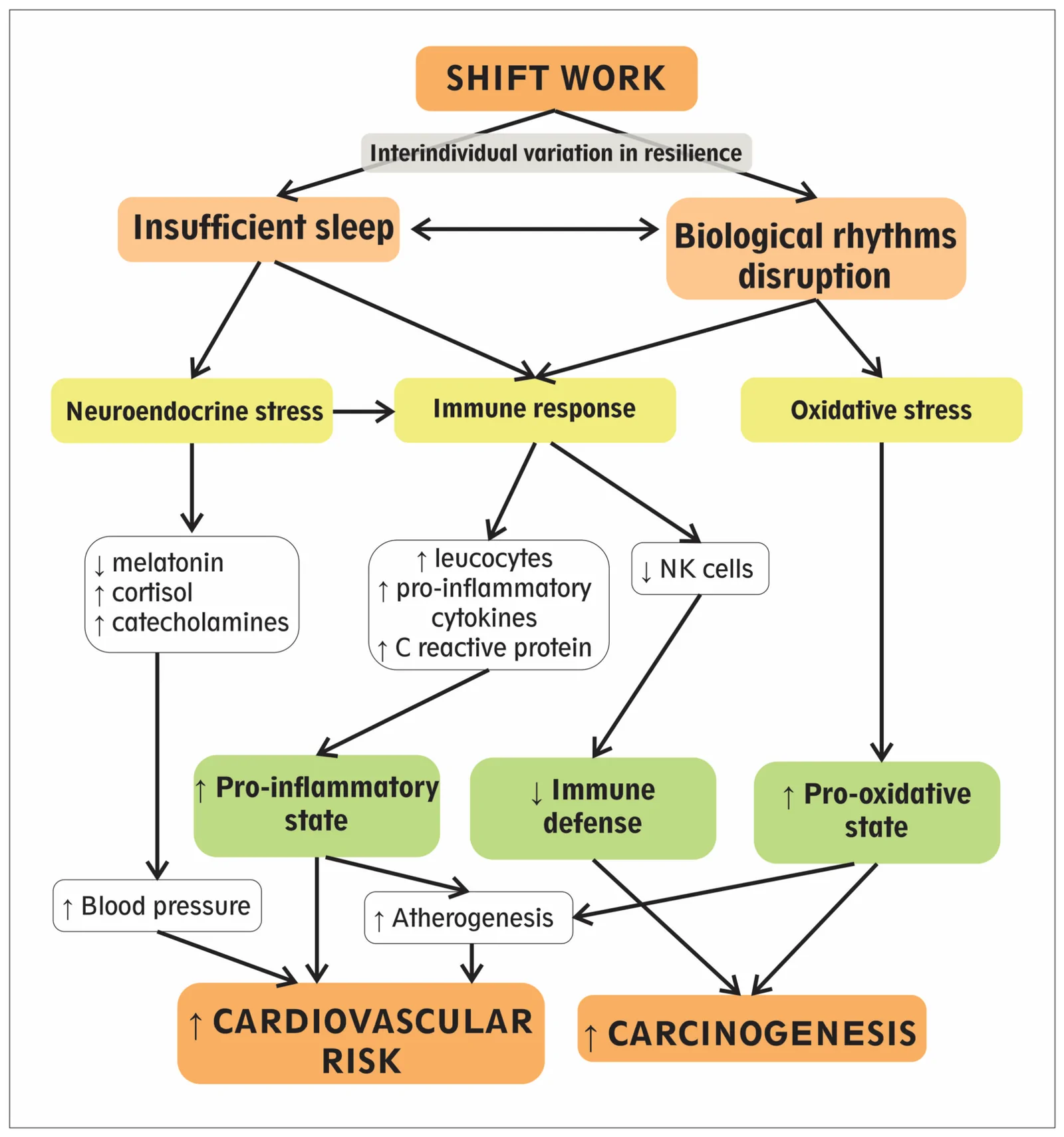
Potential physiopathological pathways by which shift work may lead to cardiovascular disease and cancer. Circadian misalignment and sleep restriction can disrupt and amplify neuroendocrine stress responses, weaken immune defenses, and promote inflammation and oxidative damage. Differences in individual vulnerability to these effects help explain the wide variability in tolerance to shift work. Adapted with permission from [28].

In addition, multiple studies have confirmed a link between shift work and cardiovascular disease. Shift workers have an approximately 20% increased risk of experiencing a cardiovascular disease event compared with day workers [74,75]. Shift work is also associated with metabolic syndrome, a key contributor to cardiovascular risk. Notably, rotating shift schedules have been identified as an independent risk factor for high blood pressure—an effect found to be even stronger than that of age or body mass index (Figure 9) [76,77,78].

In shift workers, misalignment between the body’s internal clock (circadian pacemaker) and the timing of sleep, wakefulness, and work can lead to a condition known as shift work disorder. This disorder is marked by symptoms such as insomnia, reduced total sleep time, and excessive daytime sleepiness [79]. These issues impair cognitive performance, alertness, and mood, and significantly increase the risk of accidents. While the public, media, and regulatory bodies have long pointed to speeding and alcohol consumption as the main causes of traffic accidents, it’s crucial to recognise that sleep deprivation can impair driving ability just as much as alcohol does. Psychometric studies have shown that being awake for 17–18 h affects performance to a degree comparable to having a blood alcohol concentration of 0.05 g/dL [80]. Furthermore, the combination of sleep deprivation and alcohol can have an additive negative effect on attention. Fatigue and drowsiness are estimated to contribute to 20–25% of road accidents, especially between 2:00 and 8:00 a.m., with higher rates observed on highways and monotonous routes [81].

Numerous studies on this topic have shown that a significant number of drivers regularly experience drowsiness while driving [82,83,84]. In fact, sleepy drivers are more common on the roads than drunk drivers. Our studies in Argentina further support these observations. In representative samples of public transportation drivers from the Buenos Aires Metropolitan Area, Argentina, as well as long-distance drivers operating across various regions of the country, we conducted surveys on health and working conditions and applied objective physiological assessments. These included monitoring sleep–wake patterns using actigraphy, evaluating circadian rhythms through peripheral body temperature, measuring alertness via psychomotor response to stimuli, assessing autonomic nervous system activity through heart rate variability, and evaluating stress-related endocrine responses by measuring salivary cortisol [85,86,87]. Among short-distance drivers, there was a high prevalence of work-related stress, overweight and obesity, physical inactivity, and hypertension. Sleep during the workweek was both insufficient and of poor quality, with only partial recovery on weekends. Drivers frequently experienced daytime sleepiness and showed a high risk of sleep apnea. Their weekday neurohormonal patterns reflected elevated stress levels, and a significant decline in psychomotor performance was observed during working hours, particularly during the morning shift [86,87].

Among long-distance drivers, there was also a high prevalence of cardiovascular risk factors, including overweight, physical inactivity, and smoking. Although their total weekly sleep duration was close to the recommended amount for adults, it was spread across different locations and times of day. Work schedules classified as high-risk were linked to disruptions in circadian body temperature rhythms [85].

In a subsequent study, we compared physiological and psychoaffective variables among shift workers from an Argentine oil company working under three different shift schedules. We observed that although workers assigned to all three schedules slept less than the recommended 7 h per day, those with lower sleep regularity and greater circadian disruption reported more severe insomnia symptoms, greater fatigue, reduced alertness, and higher levels of depressive symptoms. Moreover, regardless of work schedule, between 44% and 47% of participants had overweight [88]. These findings are consistent with studies conducted in Australian train drivers, where it was observed that the break timing influenced sleep duration and quality, particularly during consecutive night shifts and extended work periods [89].

Similarly, in a study of Argentine medical residents, we found that self-reported medical errors were associated with a greater proportion of daytime sleep measured by actigraphy, suggesting that circadian disruption may contribute to impaired clinical performance [90]. These findings are consistent with previous studies showing that extended work shifts increase medical errors, attentional failures, and fatigue among medical residents, whereas reducing duty hours improves both physician performance and patient safety [91,92]. However, there are other studies in which it was found that the elimination of such extended shifts did not change or worsen medical errors occurrence [93,94].

Additionally, sleep deprivation leads to allostatic overload, which can result in deleterious effects [95]. Sleep restriction to just 4 h per night has been linked to elevated blood pressure, reduced parasympathetic activity, increased evening levels of cortisol and insulin, and heightened appetite driven by increased ghrelin, a hormone that stimulates hunger, and reduced leptin, which normally suppresses appetite. Even a moderate reduction in sleep to 6 h per night raises proinflammatory cytokine levels and impairs performance on tasks requiring sustained attention, such as psychomotor vigilance tests [25,96]. In animal studies, allostatic overload results in neuron atrophy in the hippocampus and prefrontal cortex, areas involved in memory, attention, and decision-making, while neurons in the amygdala, a region that regulates fear, anxiety, and aggression, become enlarged [95]. As a result, cognitive functions such as learning, memory, and decision-making may deteriorate, accompanied by heightened anxiety and aggressive behaviour.

Beyond the well-documented effects of shift work on sleep, eating habits, and social life, gastrointestinal disorders are also very common among shift workers. The link between rotating shifts and gastrointestinal issues is multifactorial. Irregular eating patterns disrupt the synchronisation of various circadian rhythms, particularly those governing digestion and metabolism [97]. Gastrointestinal problems may arise from eating at biologically inappropriate times, leading to abnormal gastrointestinal motility and secretion patterns. Additionally, the lack of access to hot meals, especially at night when workers often rely on snacks, along with high carbohydrate intake, frequent consumption of caffeine and alcohol, and elevated tobacco use have all been proposed as contributing factors to gastrointestinal issues in shift workers [98,99,100].

Inappropriate meal timing has also been implicated in the weight gain and metabolic dysregulation associated with shift work [101]. Moreover, the evidence suggests that this misalignment is directly associated with the development of type 2 diabetes mellitus (T2DM) [102]. Several mechanisms have been proposed to explain this association, including exposure to artificial light at night and alterations in melatonin signalling. Exposure to artificial light at night, even at low intensities, has been shown to disrupt meal timing and promote weight gain, suggesting that nighttime lighting may play a significant role in the rising incidence of metabolic disorders, including T2DM [103]. One proposed link between circadian disruption and T2DM involves melatonin. Research indicates that insulin secretion is inversely related to plasma melatonin levels, and individuals with T2DM typically exhibit reduced melatonin concentrations. Laboratory studies have demonstrated that melatonin can inhibit glucose-stimulated insulin release in pancreatic cells, suggesting a direct influence on insulin function. Additionally, disturbances of circadian rhythms can impair melatonin production and negatively affect pancreatic β-cell function. Genetic studies further support this connection, revealing that mutations in melatonin receptor genes are associated with increased susceptibility to T2DM [104].

These findings suggest that disruptions in the phase, amplitude, or synthesis of melatonin may impair glucose metabolism in individuals with circadian misalignment, such as shift workers. In particular, suppression of melatonin secretion due to nocturnal light exposure could be a key contributor to the development of T2DM [103]. Therefore, effective management of T2DM may require treatments that not only target metabolic imbalances but also restore circadian rhythm integrity. In this context, melatonin, especially when combined with morning light, represents a promising chronotherapeutic approach to support metabolic health in T2DM patients.

## 11. Strategies for Circadian Entrainment in Shift Workers

The alterations caused by shift work are due to a mismatch in the circadian system of a person who hasn’t adapted to a new sleep–wake schedule. To address these issues, treatment typically involves using alternative zeitgebers to help the internal clock align with the new schedule—or, in cases like rapidly rotating shifts, to prevent the body from adjusting when it’s not needed.

There are various interventions that can be used to cause a phase shift in the circadian clock and optimize adaptation to shift work conditions. As with most pharmacological and non-pharmacological interventions, it is crucial to consider the timing within the circadian cycle when administering them.

As discussed before, the effect of light or melatonin on the circadian system can be measured by a PRC. When light is administered before the body’s core temperature reaches its lowest point, it delays the circadian rhythm; when administered after this point, it advances the rhythm. The strongest phase shifts occur when light exposure happens near this temperature minimum [39]. Carefully timed bright light can lead to phase delays of about 2.5 to 3 h per day and advances of around 1.5 to 2 h per day [37]. PRC for melatonin works in the opposite direction to that of light, although the patterns are not mirror images.

Strong evidence shows that a light stimulus, when delivered at a specific circadian phase and with enough intensity, can effectively reset the human circadian clock by pushing the circadian oscillator into a near-phaseless state—where the amplitude of the circadian rhythm drops to nearly zero. This phenomenon is known as type 0 resynchronisation [36]. In fact, when people are exposed to cycles of bright light timed to coincide with the circadian system’s peak sensitivity to light, the internal circadian rhythm’s amplitude can be significantly dampened or reduced.

One strategy that has been shown to be an effective tool to manage circadian adaptation and mitigate the negative effects of shift work is the use of bright light therapy and/or glasses that block specific light wavelengths [105]. Orange-tinted glasses have been found to block the melatonin-suppressing effects of bright light more effectively than neutral gray ones. A study conducted on police officers working seven consecutive eight-hour night shifts found that those exposed to intermittent wide-spectrum bright light at night and who wore orange-tinted glasses at sunrise showed no changes in melatonin secretion but demonstrated improved performance in a psychomotor vigilance task at night compared to the control group [106]. Moreover, night shift workers exposed to short-wavelength (blue) light during two consecutive night shifts showed improvements in subjective alertness and performance on the Psychomotor Vigilance Task [107]. Laboratory studies have also yielded promising results. In a simulated submarine lighting environment, where participants underwent four consecutive periods of simulated night work, exposure to blue-enriched and dim, blue-depleted lighting at specific times accelerated the entrainment of circadian temperature and melatonin rhythms to shift work [108]. In a similar study, participants subjected to simulated night shift work who received blue-enriched white light at times predicted to induce strong circadian delays, along with dim, blue-depleted white light for the rest of the day, slept 52 min longer and showed improvements in sleepiness and vigilance [109]. Finally, some studies have shown that inadequate light exposure during the day leads to stronger melatonin suppression and a greater phase shift response after nighttime light exposure, suggesting a protective role of daylight [110]. Although numerous studies have demonstrated beneficial effects of appropriately timed light exposure on circadian adaptation and alertness, the magnitude of these effects has varied across studies. This variability likely reflects differences in study design, including the intensity, spectral composition, duration, and timing of light exposure, as well as differences in work schedules, individual circadian phase, chronotype, and prior light history [111,112].

Nevertheless, the available evidence has been considered sufficiently robust for the Working Time Society to propose the following recommendations regarding the use of light and light-avoidance strategies to facilitate circadian adaptation in workers with non-standard schedules [105]:

- Wearing orange glasses between 6 and 9 am helps block the effects of morning bright light and facilitates circadian phase delay, which is especially important for workers commuting between 5 and 8 am.
- To readapt to daytime after night shifts, expose yourself to bright light (or natural light) in the morning or at the appropriate time according to your individual circadian phase.
- During day shifts, work near a window that allows natural daylight to promote alertness.
- Lighting interventions should consider the following factors: light spectrum (blue wavelengths have stronger effects), light intensity, duration of exposure, timing of exposure, and light history (greater daylight exposure reduces the impact of ALAN).

Additionally, it must be taken into account that there is a significant interindividual variability in how the circadian clock responds to the changing shift cycles, and so the magnitude and direction of the circadian phase shift in response to multiple consecutive night shifts is different. These findings also illustrate the substantial heterogeneity in circadian adaptation among shift workers. Moreover, it was observed that the combination of light exposure (relative to individual circadian phase) and diurnal preference explained 71% of this interindividual variability [113]. These results suggest the need for developing personalised light intervention strategies.

Not all types of light have a significant impact on the circadian clock. The non-image-forming effects of light are mediated by a class of photoreceptors known as intrinsically photosensitive retinal ganglion cells, which are particularly sensitive to short wavelengths and contain a photopigment called melanopsin [114]. Recently, a new metric has been developed to quantify the biological effects of light on non-image-forming responses: the melanopic equivalent daylight illuminance, which accounts for both light intensity and spectral sensitivity [115].

Melatonin is considered the standard example of a chronobiotic drug [116]. It is produced by a wide range of organisms—from algae to mammals—though its function varies greatly across species [29]. In humans, as previously noted, melatonin plays a key role in regulating circadian rhythms, particularly the sleep–wake cycle. Its release is driven by signals from the SCN, acting as an extension of the biological clock. The timing of melatonin secretion reflects the current state of the circadian system, indicating both its phase (how internal time aligns with external time) and its strength or amplitude. Additionally, melatonin can be seen as a chemical signal of nighttime: the longer the night, the longer it is secreted. In many animals, this secretion pattern acts as a cue for seasonal timing [25].

The chronobiotic properties of melatonin can be used to readjust the circadian phase in shift workers or those working under non-standard schedules [117]. When administered late at night or early in the morning, it can induce a phase delay, whereas administration during the day has no effect on the circadian rhythm phase [118]. There is evidence suggesting that melatonin administration (1 to 10 mg) after a night shift may increase sleep duration in shift workers [119]. Moreover, a study involving overweight nurses working permanent night shifts found that when melatonin was administered only on nights when they slept at night, it led to a reduction in circadian misalignment and body weight [120]. Although exogenous melatonin has been widely used as a chronobiotic to facilitate circadian adaptation in shift workers, the magnitude and consistency of its phase-shifting effects remains an area of ongoing investigation, as they depend on several factors, including the timing of administration relative to the endogenous circadian phase, dose, formulation, and interindividual variability [33]. Consequently, the effectiveness of melatonin in real-world shift-work settings is likely to vary across individuals and work schedules, highlighting the need for personalised chronotherapeutic strategies.

**Figure 10:**
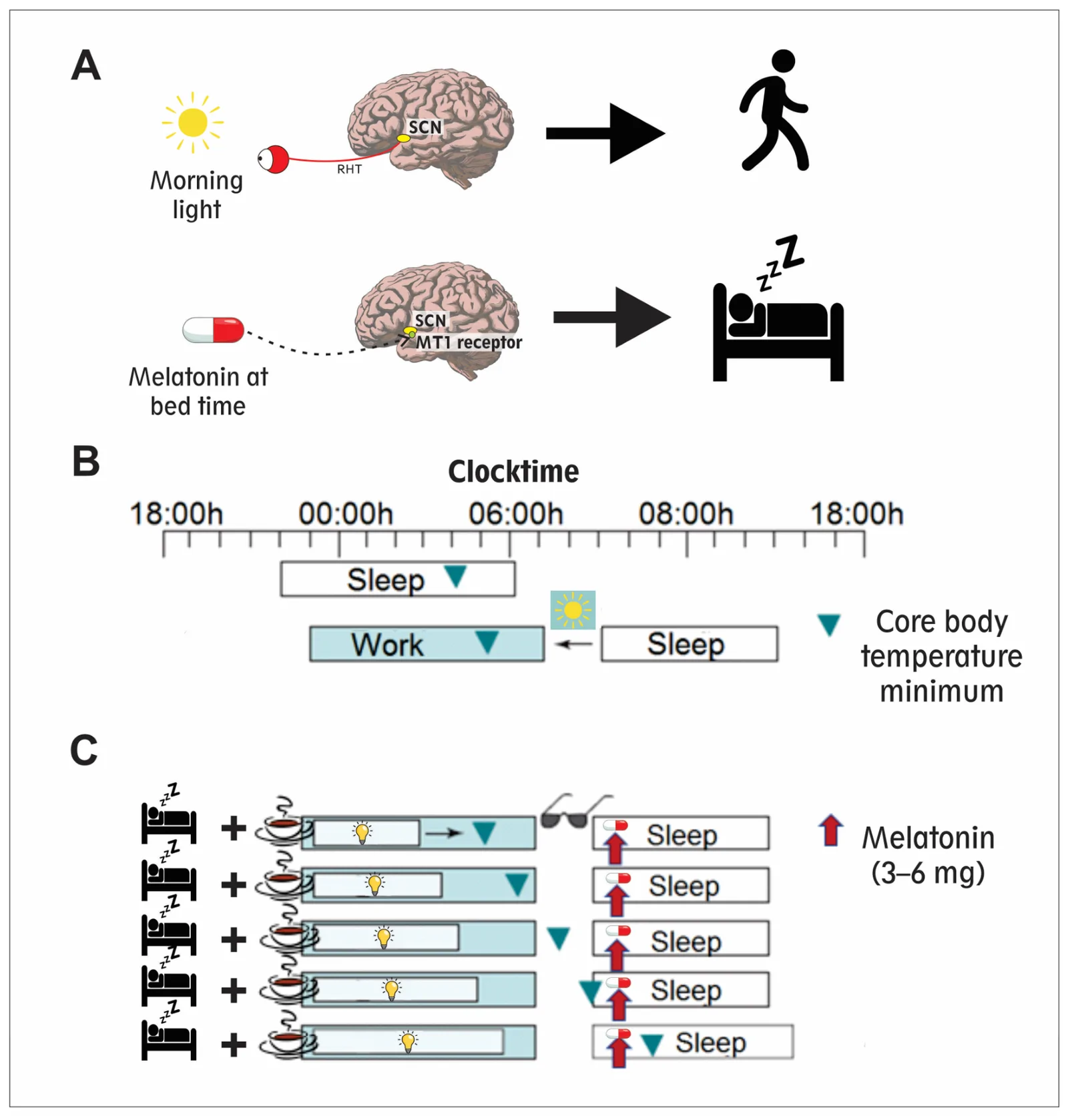
Strategies to synchronise the circadian clock of shift workers. (**A**) Morning light exposure and evening melatonin administration provide a strong synchronising signal and enhance the amplitude of the sleep–wake cycle. These are natural tools to restore proper rhythmicity of this cycle. (**B**) This is an example of a night worker on a 5-day night shift. To ensure restorative daytime sleep, the minimum of core body temperature needs to be shifted towards the sleep period. Light exposure and activity during the night shift cause a phase delay, which is partly counteracted by sunlight during the commute home. (**C**) Gradually increasing periods of bright light during the first part of the night shift promote a phase delay, which can be maintained by wearing dark glasses on the way home and taking melatonin before sleeping. Melatonin administration before daytime sleep helps induce sleepiness and supports phase shifting. Additionally, planned 30-min naps before or during the shift, combined with caffeinated beverages, can reduce sleepiness and improve alertness at work. Modified and adapted with permission from [25].

Figure 10 illustrates a typical scenario for someone working five consecutive night shifts. To align the body’s internal clock with daytime sleep, the core body temperature minimum should be shifted by exposing the individual to bright light during the early part of the night shift and using dark goggles during the commute home to block morning light. Taking melatonin (3–6 mg) before going to sleep during the day can promote sleepiness and help shift the circadian phase [121]. Strategic 30-min naps either just before or during the shift, along with caffeine, can reduce drowsiness and boost alertness while on the job. Additionally, the Food and Drug Administration from the USA Government has approved the wake-promoting medications armodafinil and modafinil to treat excessive sleepiness in individuals diagnosed with shift work disorder.

In managing shift work disorder effectively, organizational-level strategies also play a crucial role. Three key interventions have been proposed: (a) adopting fast instead of slow rotation of shifts (rotating from one shift to another once every few days), (b) using forward (clockwise) rather than backward (counterclockwise) rotation, and (c) allowing workers to self-schedule their shifts [79]. Research suggests that fast-rotating schedules are less harmful because they reduce the amount of time workers spend in a misaligned circadian state. Workers also tend to prefer forward rotation, likely because the human biological clock runs slightly longer than 24 h. Additionally, longer shifts can lead to more extended periods off duty. Given the wide variation in individual responses to shift work, a flexible, personalised approach may be most effective. Ultimately, the primary objective is ensuring workers get at least 7 h of sleep within each 24-h period. Finally, the implementation of Fatigue Risk Management Systems may be very useful to improve sleep and prevent and detect fatigue [122].

Exposure to light at night, even at low intensities, has been shown to affect the timing of food intake and contribute to weight gain, highlighting the role of artificial lighting in the growing incidence of metabolic disorders [103]. When food is limited in quantity and available only at specific times, it can serve as a cue to synchronise certain brain structures that regulate various circadian rhythms [123]. This can lead to a dissociation between rhythms governed by light via the SCN and those regulated by food, through what is known as the food-entrainable oscillator (FEO). In animals receiving a single daily meal with fewer calories than needed, physiological rhythms begin to align with the feeding schedule. Indicators such as motor activity, gastrointestinal movement, digestive enzyme activity, and plasma cortisol levels tend to increase one to two hours before the expected mealtime. Interestingly, this anticipatory behaviour is driven by a pacemaker separate from the SCN, as it persists even in animals with SCN damage [123]. When food is plentiful, the SCN, entrained by light, controls the body’s circadian rhythms. However, under conditions of food scarcity and restricted access, a secondary oscillator (FEO) takes over the regulation of certain rhythms to optimize food use, while other processes remain under the influence of the SCN. In a laboratory protocol with humans, meal timing was shown to affect the phase of the plasma glucose rhythm, average glucose concentration, and circadian gene expression in adipose tissue, without altering sleep parameters [124]. In conclusion, another non-photic stimulus that may be used to phase-shift the human circadian clock could be food.

A situation comparable to shift work is experienced by individuals who travel across multiple time zones. Jet lag, or flight dysrhythmia, encompasses a range of symptoms including daytime fatigue, reduced alertness, insomnia during the night, appetite loss, low mood, diminished motor coordination, and decreased cognitive performance [125]. These symptoms arise due to a temporary mismatch between the body’s internal circadian clock and the external time cues, caused by rapid transmeridian travel. The severity of jet lag depends on the number of time zones crossed and the direction of travel. Generally, eastward travel makes it harder to fall asleep, while westward travel more commonly disrupts staying asleep.

In summary, jet lag and shift work disorders stem from a common underlying cause, namely circadian misalignment, and their management shares several key strategies. In both cases, three factors play a critical role: (1) appropriately scheduling sleep, (2) resetting the internal clock using light exposure and/or chronobiotic agents like melatonin, and (3) administering stimulants or wake-promoting drugs when necessary.

## 12. Current Limitations and Future Perspectives

Although substantial progress has been made in understanding the mechanisms underlying circadian regulation and the physiological consequences of circadian disruption, several important questions remain unsolved. The evidence supporting many chronotherapeutic interventions is still heterogeneous because of differences in study design, participant characteristics, work schedules, outcome measures, and intervention protocols. In particular, further studies are needed to determine the optimal timing, intensity, duration, and combination of light exposure and melatonin administration for different shift-work schedules and chronotypes. Likewise, the mechanisms underlying the chronobiotic effects of melatonin and the relative contribution of central and peripheral clocks to circadian adaptation remain areas of active investigation.

In addition, more research is needed to better characterise the PRCs of the major outputs of the circadian system. A deeper understanding of these PRCs would facilitate the design of evidence-based chronotherapeutic interventions capable of selectively shifting the phase of both central and peripheral clocks. Such interventions, together with personalised approaches that consider individual variability in circadian phase, chronotype, and light exposure history, may improve adaptation to shift work while reducing its adverse effects on health, safety, and well-being.

Ultimately, translating advances in circadian biology into individualised chronotherapeutic strategies represents one of the major challenges and opportunities for improving the health, safety, and quality of life of people working under non-standard schedules.

## Data Availability

No new data were created or analyzed in this study. Data sharing is not applicable to this article.

## Abbreviations

The following abbreviations are used in this manuscript:

ALAN	Artificial light at night
AMPK	5′ adenosine monophosphate-activated protein kinase
aMT6s	6-sulfatoxymelatonin
bHLH-PAS	Basic helix–loop–helix–Period–Arnt–Single-minded
Bmal1	Brain and muscle ARNT-like 1
CBTmin	Minimum of the core body temperature rhythm
CCGs	Clock-controlled genes
CK	Casein Kinase
Cry	Cryptochrome genes
DLMO	Dim light melatonin onset
DMH	Dorsomedial hypothalamus
FEO	Food-entrainable oscillator
GABA	Gamma-aminobutyric acid
Per	Period genes
PRC	Phase response curve
PVN	Paraventricular nucleus
RHT	Retinohypothalamic tract
RORE	Retinoic acid-related orphan receptor response elements
SCN	Suprachiasmatic nuclei
T2DM	Type 2 diabetes mellitus
